# Diagnosis and treatment of benign adipocytic tumors in children

**DOI:** 10.1007/s00383-022-05248-2

**Published:** 2022-10-20

**Authors:** Laura Daniela Pérez Daza, Laura Camila González Villarreal, Laura Camila Sánchez Rodríguez, Iván Darío Molina Ramírez, Edna Margarita Quintero Canasto

**Affiliations:** 1 Universidad Nacional de Colombia, Fundación Hospital Pediátrico la Misericordia, Av. Caracas # 1-65, 111411 Bogotá, DC Colombia; 2 Universidad Nacional de Colombia, Carrera 45 # 26-85, 111321 Bogotá, DC Colombia; 3 Department of Surgery, Fundación Hospital Pediátrico la Misericordia, Universidad Nacional de Colombia. Av, Caracas # 1-65, 111411 Bogotá, DC Colombia; 4 Fundación Hospital Pediátrico la Misericordia, Av. Caracas # 1-65, 111411 Bogotá, DC Colombia

**Keywords:** Adipose tissue neoplasms, Lipoma, Lipoblastoma, Child

## Abstract

**Purpose:**

To describe demographic, clinical, diagnostic and therapeutic aspects of pediatric patients with benign adipocytic tumors admitted to a high complexity teaching hospital from 2007 to 2021.

**Methods:**

Retrospective observational descriptive study. Patient information was retrieved from clinical records. A descriptive analysis was carried out for qualitative data and frequencies were calculated for quantitative data.

**Results:**

76 patients were included with a mean age of 7.5 years old where 60.5% were boys. The main symptom was a mass (73.7%) mostly found in the lower limbs (23.6%). Congenital birth defects were identified in 48.6% of the cases. Preoperative imaging was available in 78.9% of the patients allowing characterization of lesions or differential diagnosis. The therapeutic goal was resection with negative margins, which was feasible in all cases except for one case. The histopathological diagnosis was lipoma in 68.4% of the cases followed by lipoblastoma in 13.1%. The mean follow-up period was 17.9 months. 79.7% of the patients were asymptomatic at their last out-patient visit.

**Conclusion:**

Benign adipocytic tumors constitute a wide spectrum of lesions, which involve diverse anatomic segments from the neural axis to the inguinoscrotal region. The present work contributes to the general understanding of the clinical presentation and differential diagnosis for these infrequent neoplasms.

## Introduction

Adipocytic neoplasms account for less than 10% of soft tissue tumors in the pediatric population. They are histologically distinct from adult tumors and can be associated with genetic syndromes [1, 2]. There is a wide group of benign lesions which include lipomas, lipoblastomas, hibernomas, angiolipomas and myolipomas. However, there are borderline lesions including atypical lipomatous tumors and malignant lesions, such as liposarcoma [3]. Some of these malignant variants can be difficult to classify due to the presence of myxoid components, which are characteristic of benign types [4]. Recent developments attempt to describe genetic features of these neoplasms; nevertheless, they remain poorly understood [5].

Differential diagnosis of soft tissue masses in pediatric patients requires knowledge of the patient’s age, time of onset, location, variations in size, cutaneous changes, and comorbid conditions. Taking this into account, most adipocytic tumors are present as superficial, usually painless, masses located in the limbs. Ultrasound (US) is the first-line test for diagnostic evaluation given its availability, low cost and lack of ionizing radiation and exposure to contrast agents [6]. However, for deep lesions or those which cannot be properly evaluated with sonography, magnetic resonance (MR) is the imaging diagnostic test of choice [7]. Plain X-rays and computed tomography (CT) are limited, except for cases with suspicion of bone compromise [6]. During imaging evaluation, it must be taken into account that other tumors can also include adipose tissue, such as fibroblastic tumors and vascular anomalies. Macroscopic fat identification can narrow the differential diagnosis with a high possibility of a benign etiology [7, 8].

On microscopic examination benign lipomatous tumors resemble white adipose tissue with variable differentiation grades [1]. The therapeutic principle with excellent prognosis is a negative margin resection. Nonetheless, recurrence associated with incomplete resection has been documented in 13–46% of the cases. Despite being a spectrum of infrequent pathologies, the adequate diagnostic approach of lipomatous tumors in children constitutes a fundamental competence of the pediatric surgeon in the evaluation of soft tissue masses [7]. The goal of this study was to present a pediatric case series describing the clinical, radiological, and pathological features and treatment options of benign adipocytic tumors. In addition, we aimed to characterize the local epidemiology of pediatric adipocytic tumors due to the lack of general information.

## Methods

A retrospective descriptive observational study was designed with the approval of the Institutional Ethics Committee (Act No. 45 382–21). Data were retrieved from the Fundación Hospital Pediátrico la Misericordia pathology laboratory database, Bogotá D.C., Colombia. 107 records with histopathological diagnosis of benign lipomatous tumors were identified between January 2007 and July 2021. Cases with findings that were compatible with variants of “lipoma” and “lipoblastoma” were included for a total of 76 patients. 31 cases were excluded. 8 of them were duplicate registries from a same patient. 23 were referral cases from a regional institution to our Pediatric Pathology Laboratory, therefore clinical records were unretrievable.

Demographic and clinical data including age, sex, comorbidities, main complaint, associated symptoms, mass location, diagnostic imaging, surgical approach, and histopathological diagnosis were obtained by reviewing medical records. A follow-up period was accurately recorded for 70 cases and was defined as the time in months beginning from diagnosis until the last out-patient visit. Due to the nature of the study, no statistical analysis was performed. Nonetheless, descriptive statistics including mean, median and range were calculated for quantitative data and frequencies expressed as percentages for qualitative data.

## Results

**Fig. 1:**
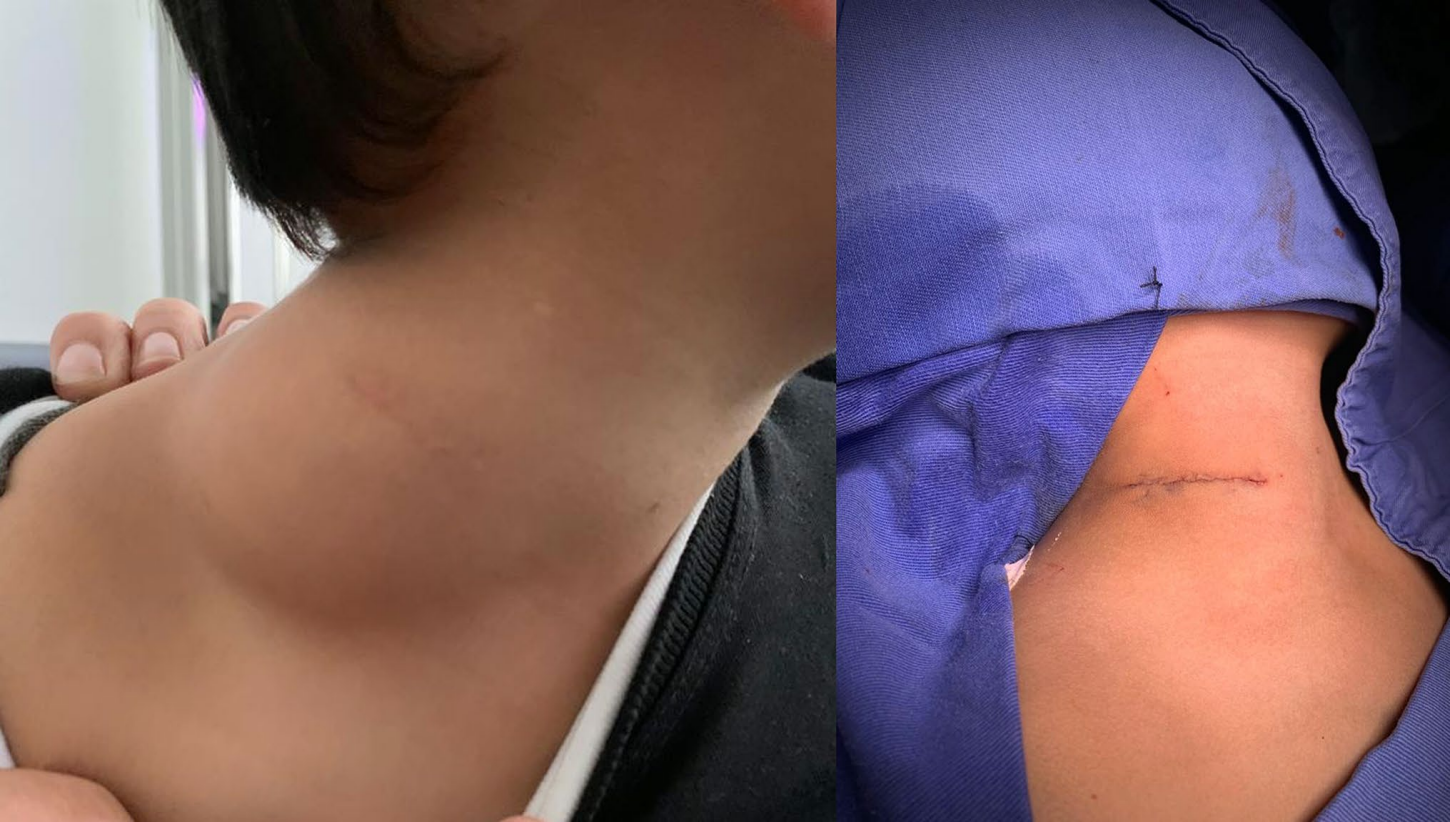
Toddler with progressively growing neck soft tissue mass with history of previous resection (left). Appearance after second surgical resection (right)

The mean diagnosis age was 7.5 years (range 0.4–17 years, median 8), where 60.5% of the population were male and 39.4% were female, with a male:female ratio of 1.5:1. The most frequent symptom was a mass (*n* = 56, 73.7%), followed by pain (*n* = 9, 11.8%), deformity (*n* = 3, 3.9%) and others such as cough, gait instability, joint edema, dysraphism and myoclonic crisis (Fig. 1). In six cases (7.8%) inguinoscrotal syndrome was the initial presentation. Two cases (2.6%) were documented as incidental findings. One of them was a patient with a dysmorphic syndrome characterized by hydrocephalus and cleft palate, who underwent surgical resection of a suspected lower limb skin tag. The second case corresponded to a child with occult dysraphism and lumbar tethered cord syndrome who was initially asymptomatic.

The most frequent findings were observed in the lower limbs (*n* = 18, 23.6%), followed by dorsolumbar soft tissues (*n* = 9, 11.8%), inguinal region (*n* = 7, 9.2%), upper limbs (*n* = 7, 9.2%), spinal canal (*n* = 7, 9.2%), neck (*n* = 5, 6.5%), chest wall (*n* = 4, 5.2%) and retroperitoneal location compromising renal parenchyma (*n* = 3, 3.9%). Other less frequent locations were the gluteal region (*n* = 3, 3.9%), perineum (*n* = 3, 3.9%), abdominal wall (*n* = 3, 3.9%), frontal region (*n* = 3, 3.9%), scrotum (*n* = 2, 2.6%), oral cavity (*n* = 1, 1.3%) and peritoneal cavity compromising the omentum (*n* = 1, 1.3%).

In 47.3% of the cases congenital anomalies were a main comorbidity, among which neural tube closure defects with tethered cord syndrome (16.6%), epileptic syndromes mostly associated with tuberous sclerosis (13.8%) and anorectal malformations (5.5%) were the most frequently described. Two procedures were carried out on a patient with lipoblastomatosis who was previously diagnosed with Proteus syndrome.

**Fig. 2:**
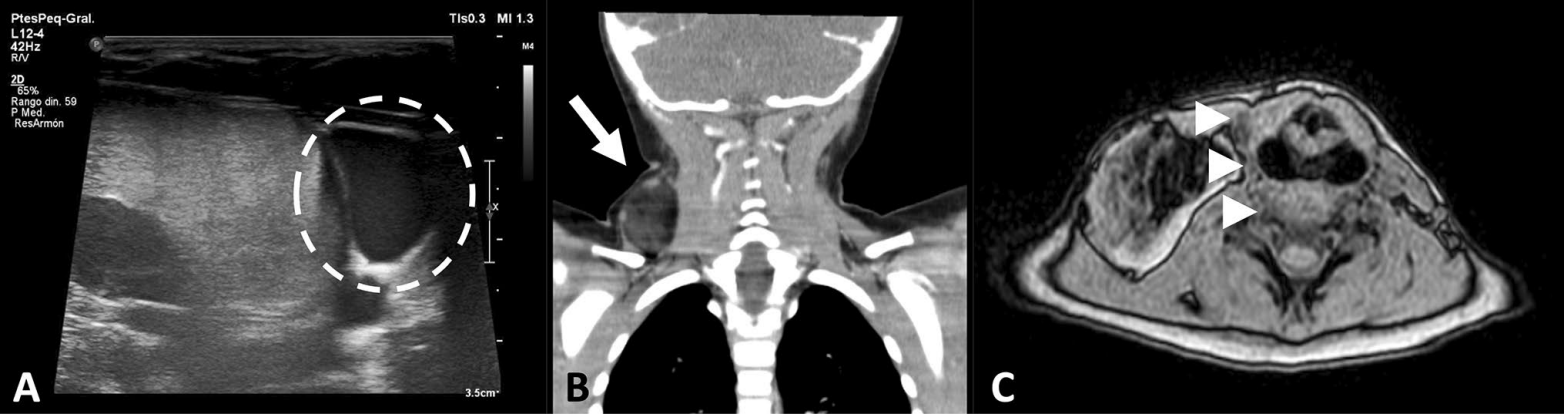
Imaging modalities available for lipomatous tumors. **A** Hypoechoic, well-circumscribed soft tissue lesion on ultrasound (dotted line). **B** Hypodense, encapsulated mass on coronal CT with heterogeneous contrast enhancement (white arrow). **C** Heterogeneous mass with fat signal intensity on axial MR, which displaces adjacent structures (arrow heads)

Pre-procedural imaging was performed in 78.9% of the population studied. The first diagnostic test was an US, which was performed in 32.8% of the cases, 21% had a CT scan and 51.3% had a MR imaging. On 38.9% of the cases two imaging tests were performed, while only two cases had the three tests performed (3.3%). Most lesions evaluated by sonography described solid, hypoechogenic masses, with well-defined lobulated margins sparing muscular structures, some of which displayed intralesional longitudinal echogenic images and absence of flow on Doppler US mode, except for vascularized masses, such as angiolipomas (Fig. 2A). Cases with diffuse compromise were described as subcutaneous fat of variable thickness without a defined mass.

Focal, well defined, hypodense lesions with heterogeneous contrast enhancement were described by CT characterization. Additionally, these lesions included irregular septa and in most cases exerted a compressive effect on neighboring structures (Fig. 2B). On the other hand, round, heterogeneous masses with fat signal intensity and contrast enhancement of vascularized portions were defined through MR imaging (Fig. 2C). Cases with diffuse compromise, such as lipomatosis displayed an increase in subcutaneous fat thickness; however, delineation of lesion margins was not possible.

Suggested differential diagnoses as determined by preoperative imaging included lymphatic vascular malformations and other neoplasms, such as renal cell carcinoma, hemangioma, schwannoma and chondroma. Most cross sectional studies were performed for deep lesions with compromise of thoracic or abdominal cavity, muscle and joint compartments, and those located within the central nervous system. In contrast, US was performed on patients with superficial lesions or during admission as part of the study of abdominal pain or inguinoscrotal syndrome.

**Fig. 3:**
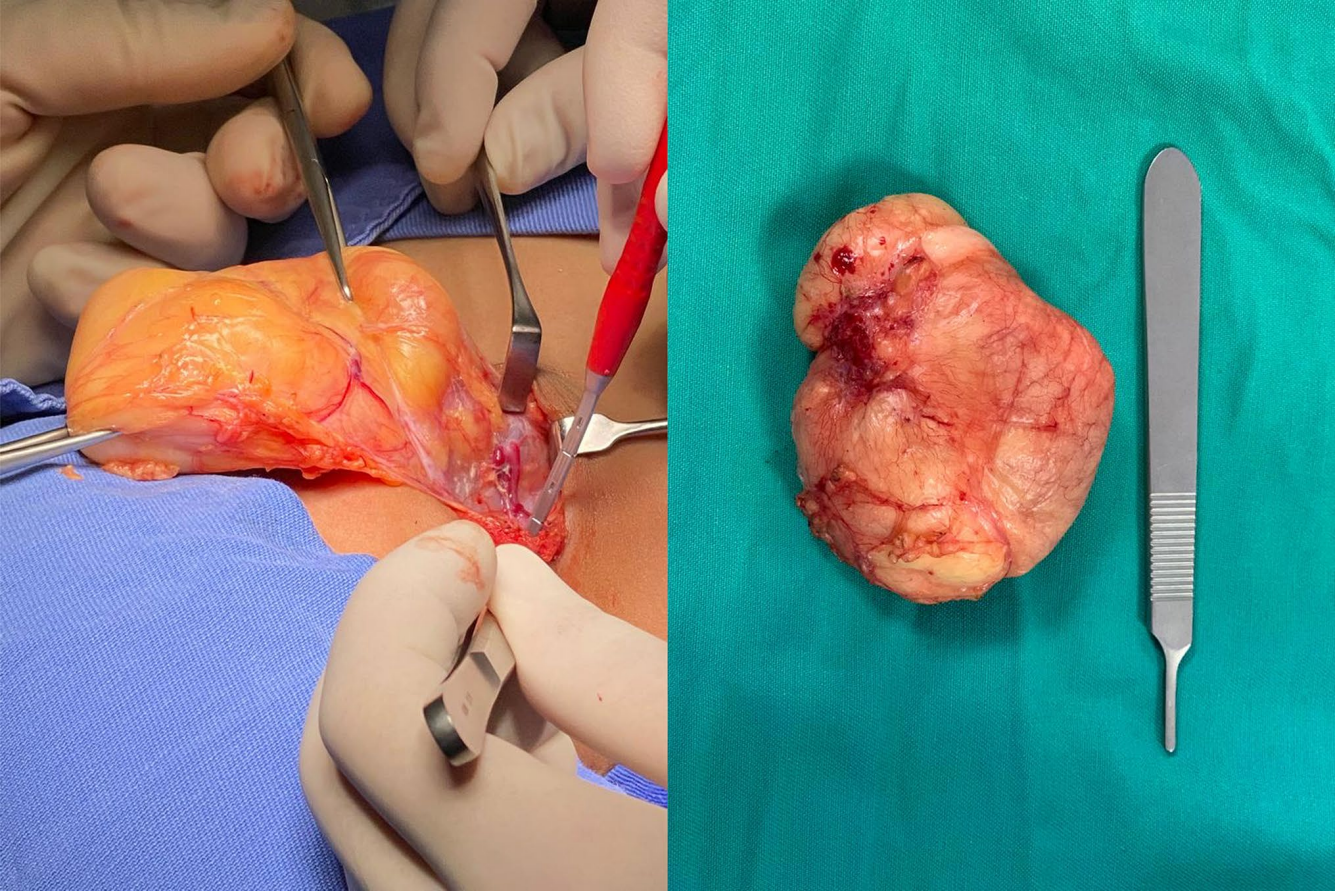
Negative margin resection of a lipoblastoma of the neck in a toddler (left). Macroscopic appearance of surgical specimen characterized by yellow and lobulated surface with a thin fibrous capsule (right)

The surgical approach was variable based on the myriad of benign adipocytic tumor clinical presentations. The therapeutic goal was complete resection with free margins (Fig. 3), which was feasible in all cases except for a patient with syndromic lipoblastomatosis. Complication rate was 10.5%. Two cases of cerebrospinal fluid fistula, one hematoma, one deep surgical site infection, one filum terminale lesion, one superficial wound dehiscence, one self-limited postsurgical bleeding case and one pleural rupture requiring chest tube placement were documented. Two recurrences were described (2.8%) requiring reoperation during the subsequent year, one of which corresponded to the aforementioned syndromic lipoblastomatosis case. Moreover, the mean follow-up period was 17.9 months (range 0.25–132, median 5).

**Fig. 4:**
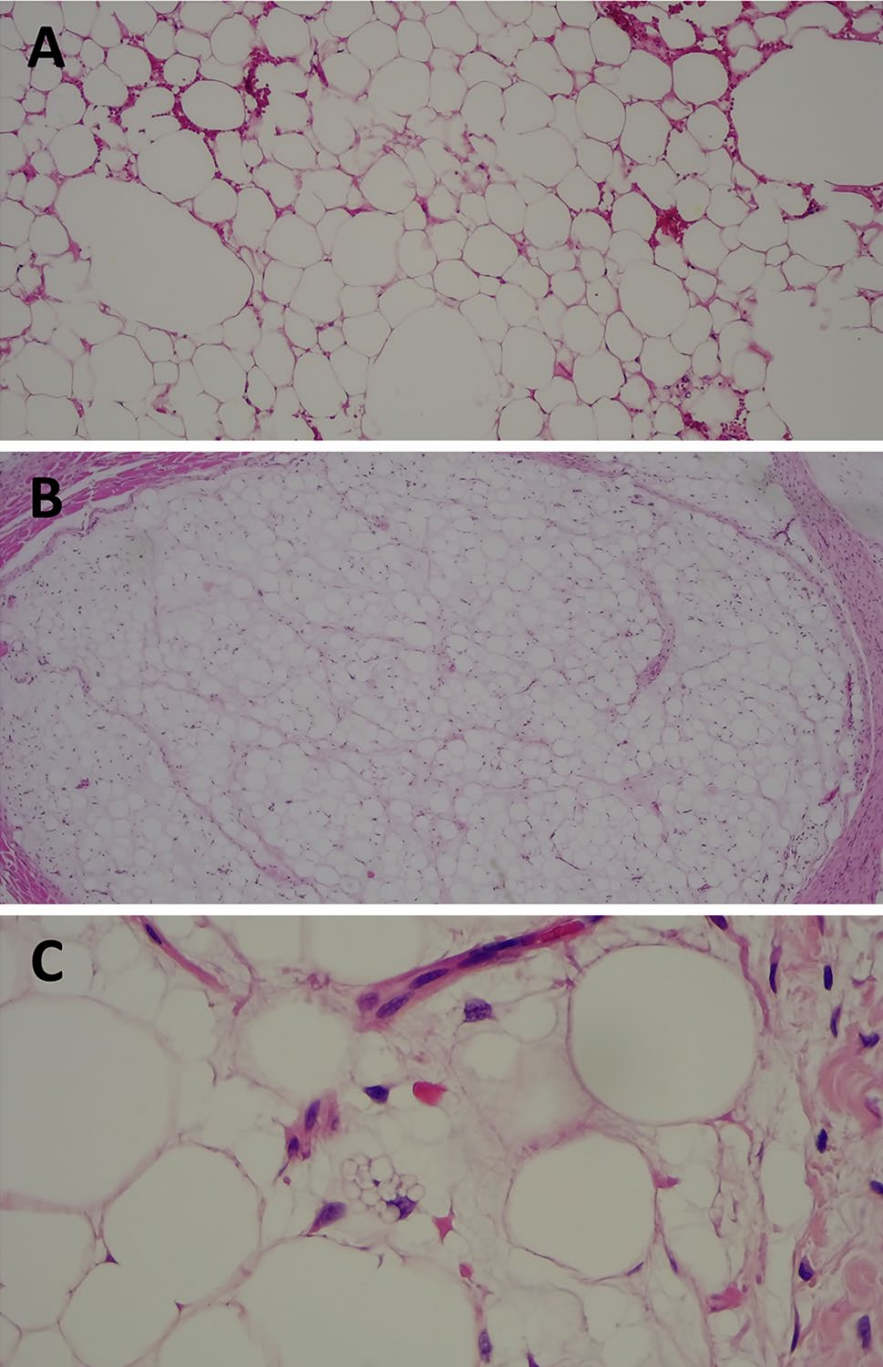
**a** Mature adipocytes in lipoma (H&E stain, 10x). **b** Lipoblastoma (H&E stain, 10x). **c** Cells with variable differentiation grades in lipoblastoma (H&E stain, 40x)

Tumor size varied between 0.5 and 20 cm (mean 5.1 cm, median 4.8). In all age groups the main histopathological diagnosis was lipoma (*n* = 51, 67.1%). On microscopic evaluation these lesions were characterized by the presence of mature adipocytes arranged in lobules separated by mature fibro-connective tissue strands, with myxoid stromal foci and capillary vascular proliferation (Fig. 4A). One of them displayed arborescent disposition and another presented bone chondroid metaplasia with hematopoiesis. The second most frequent diagnosis was lipoblastoma (*n* = 10, 13.1%). On microscopic evaluation these lesions were characterized by the presence of cells with variable differentiation grades, including prelipoblasts, lipoblasts, preadipocytes, multivacuolated adipocytes and mature adipocytes (Fig. 4B, C). The remaining cases were compatible with angiolipoma and angiomyolipoma (*n* = 9, 11.8%), fibrolipoma (*n* = 3, 3.9%) and diffuse lesions including lipomatosis (*n* = 1, 1.3%) and lipoblastomatosis (*n* = 1, 1.3%).

## Discussion

Our cohort agreed with previously published studies regarding the mean age of adipocytic tumor presentation [2], as well as gender distribution with male predilection, as described for multiple lipomas, angiolipomas and lipoblastomas [1, 9]. The age of onset was fundamental in this study because lipoblastomas tend to have a greater incidence in children under the age of three [6]. The most frequent clinical presentation was a painless soft tissue mass, with variable adipose content based on the histological subtype [7]. Interestingly, angiolipomas can be painful and have a firmer consistency due to their vascular component [1].

Depending on their location, adipocytic tumors can be associated with other manifestations such as dysraphism [2], abdominal pain or distension and bowel obstruction with intra-abdominal lesions [10, 11] or inguinoscrotal syndrome, as observed in this case series. Two patients presented anorectal malformations, one of them with a perineal lipoma and the other with a lipoma of the filum terminale, similar to previous reports [2]. The most frequently described locations are trunk and extremities. Nevertheless, there are several reports describing lesions with compromise of scrotum, inguinal region, axilla, neck, parotid region, retroperitoneum, mediastinum, omentum [9, 12] and mesentery [10, 11].

One patient from our cohort underwent multiple resections for lipoblastomatosis associated with Proteus syndrome. This condition is characterized by diffuse soft tissue overgrowth and can be associated with other disorders, such as tuberous sclerosis [1]. Other diseases likely to develop lipomatous tumors include Cowden, Gardner and Richards, Bannayan-Riley-Ruvalcaba, Robinow and Clove syndromes, as well as type 1 neurofibromatosis [2]. Hibernoma, which is a tumor derived from brown adipose tissue can be found in patients with type 1 multiple endocrine neoplasia syndrome [5]. However, no such cases were identified in our cohort.

For superficial lesions US was the diagnostic tool of choice in contrast with cross sectional imaging studies, which aimed to characterize masses with compromise of deep tissue compartments and body cavities. In most cases diagnosis was accurately determined by imaging; however, some reports required differential diagnoses, such as lymphatic malformations or alternative neoplasms, which can also contain adipose tissue. Furthermore, in this series an important number of lesions were identified as an intraoperative incidental finding in unrelated pathologies, such as testicular torsion or spermatic cord hydrocele. Even though CT imaging has limited applications for soft tissue evaluation, such as lipomatous tumor diagnosis [6], 21% of our patients had this imaging test performed to characterize deep masses or suspected bone compromise [7, 8].

In this series, most complications were related to factors associated with the resection site, unlike other cohorts which did not describe complications. In the present study reported recurrence rate was low, as had been described in the literature for lipomas, corresponding to less than 5% of the cases [12]. However, for lipoblastomas they can range from 9 to 46% [1, 10] and up to 80% in patients with incomplete resections [9]. Some locations, such as inguinal region or intramuscular tumors can be associated with greater recurrence rates [1, 2].

The mean tumor size was variable when compared to previous studies [9]. However, this could be attributed to the diverse distribution of locations, with a tendency to smaller diameters in superficial lesions (< 5 cm) in contrast with greater sizes in deep lesions (> 5 cm), although this is a subjective appreciation also described in other cohorts [6, 7]. In agreement with the literature the most frequent histopathological subtype was lipoma [1, 8], followed by lipoblastoma [4]; nonetheless, with a lower incidence in comparison with other reports which describe this subtype in up to 30% of the cases [6].

The present study aimed to report the local epidemiology of benign adipocytic tumors through the description of one of the largest case series to date centered on the clinical presentation, comorbid conditions, histopathological diagnosis, and complications associated with surgical management. The focus on benign pathology was motivated by the increasing diagnosis of lesions compatible with lipoblastoma in our setting. The results of this study support a higher incidence of lipomas in all age groups, unlike previously published literature that reports a higher frequency of lipoblastomas in the group of children under three years of age [6].

One of its limitations was the follow-up period of less than 5 years, which is the time-period usually recommended by some authors to increase the probability of identifying late recurrences [9]. Another limitation was the lack of characterization of immunohistochemical and molecular profiles, which has been thoroughly outlined in other studies, allowing diagnostic confirmation and prognostic prediction in selected cases [1, 2, 4, 5, 9–11]. Genetic consultation is, in fact, one of the most promising tools in the study of soft tissue tumors, including adipocytic neoplasms. Advances in molecular diagnostic techniques have enabled the identification of potential therapeutic targets for the management of these tumors.

For instance, 12q14–15 and 6p21 loci alterations have been shown to result in overexpression of HMGA2 and HMGA1 in pediatric lipomas. Similarly, it has been possible to characterize the overexpression of PLAG1 related to 8q11 locus alterations in lipoblastomas. The identification of these molecular patterns, as well as the diagnosis of other associated syndromes could have important therapeutic and prognostic implications in patients with adipocytic neoplasms in the future [5].

## Conclusions

Local epidemiology of benign adipocytic tumors in children in our setting is similar to that previously reported in the literature; yet we identified a lower proportion of lipoblastomas. Additionally, to properly identify late recurrences a longer follow-up period is needed. Furthermore, tumor molecular characterization is relevant for establishing accurate diagnosis of atypical disease, unfortunately this technology is not readily available for routine use in the local practice (Table 1).

**Table 1:** Demographic, clinical, radiological, pathological and therapeutic features of benign adipocytic tumors in children

Characteristic	*N* = 76 (%)
Mean age at diagnosis (range)	7.5 years old (0.4–17)
Sex	
Male	46 (60.5)
Female	30 (39.4)
Symptoms	
Mass	56 (73.7)
Pain	9 (11.8)
Inguinal hernia	6 (7.8)
Deformity	3 (3.9)
Incidental finding	2 (2.6)
Location	
Lower limbs	18 (23.6)
Dorsolumbar soft tissues	9 (11.8)
Inguinal region	7 (9.2)
Upper limbs	7 (9.2)
Spinal canal	7 (9.2)
Neck	5 (6.5)
Chest wall	4 (5.2)
Retroperitoneum	3 (3.9)
Gluteal region	3 (3.9)
Perineum	3 (3.9)
Abdominal wall	3 (3.9)
Scrotum	2 (2.6)
Oral cavity	1 (1.3)
Associated anomalies	36 (47.3)
Neural tube closure defects	6 (16.6)
Epileptic syndromes	5 (13.8)
Anorectal malformations	2 (5.5)
Proteus syndrome	1 (2.7)
Other	14 (38.8)
Preoperative imaging	60 (78.9)
Ultrasound (US)	25 (32.8)
Computed tomography (CT)	16 (21.0)
Magnetic resonance (MR)	39 (51.3)
Two modalities	23 (38.9)
All modalities	2 (3.3)
Completeness of resection	
Positive margins	1 (1.3)
Negative margins	75 (98.7)
Mean tumor size	5.1 cm (0.5–20)
Histopathological diagnosis	
Lipoma	51 (67.1)
< 3 years old	14 (27.5)
> 3 years old	37 (72.5)
Lipoblastoma	10 (13.1)
< 3 years old	3 (30)
> 3 years old	7 (70)
Diffuse disease	2 (2.6)
Other	13 (17.1)
Complications	8 (10.5)
Cerebrospinal fluid fistula	2 (25)
Hematoma	1 (12.5)
Deep surgical site infection	1 (12.5)
Filum terminale lesion	1 (12.5)
Wound dehiscence	1 (12.5)
Self-limited bleeding	1 (12.5)
Pneumothorax	1 (12.5)
Recurrence rate	2 (2.8)
Mean follow-up time (range)	17.9 months (0.25–132)
